# A qualitative study on the transition to full-scope Medi-Cal among older Latino patients in California

**DOI:** 10.1186/s12913-025-13961-6

**Published:** 2025-12-31

**Authors:** Annie Ro, Celina Morales, Connie Valencia

**Affiliations:** 1 Joe C. Wen School of Population and Public Health, University of California, Irvine, 856 Health Sciences Quad Suite 3600, Irvine, CA 92697-3957 USA; 2 Sustainability Solutions Community Engagement Fellow, USC Equity Research Institute (ERI), 1149 S. Hill St Suite H340, Los Angeles, CA 90015 United States of America

**Keywords:** Medicaid expansion, Older adults, Latino immigrants, California

## Abstract

**Background:**

In May 2022, California became the first state to extend full-scope Medi-Cal coverage to low-income residents aged 50 and older, regardless of immigration status. This study explored the older adult Medi-Cal expansion among newly enrolled undocumented patients in Southern California, focusing on barriers and facilitators to enrollment, continuity of care, healthcare utilization, and changes in health status.

**Methods:**

We examined patient perspectives on the Medi-Cal transition using both focus groups and interviews collected between April and December 2023. We collected data from 12 undocumented participants from two Southern California Federally Qualified Health Centers (FQHCs) who became eligible for Medi-Cal with the older adult expansion. We conducted a thematic analysis combining deductive and inductive techniques and developed a coding scheme around medical care before Medi-Cal expansion, participants’ experiences with enrollment process, and their health care experiences after the policy change.

**Results:**

We found that most patients were able to enroll in Medi-Cal successfully, but nearly all relied on social workers or health clinic staff to assist them. Some participants who were enrolled in a local health coverage program that pre-dated Medi-Cal expansion had continuity in their primary health care experiences. Others with more episodic care experienced improvement in health care costs. All patients shared increased feelings of security and were more willing to seek out health care with full-scope Medi-Cal.

**Conclusions:**

Our exploratory findings highlight the relative success in Medi-Cal enrollment while also calling attention to potential differences in the policy’s impact based on pre-expansion health care availability, as well the vital importance of professional enrollment support.

**Supplementary Information:**

The online version contains supplementary material available at 10.1186/s12913-025-13961-6.

## Background

While Medicaid’s lawful immigrant requirement restricts eligibility to citizens and certain qualified non-citizens (e.g., green card holders with longer than 5 years US duration, refugees, asylees), California has used state funds to expand Medi-Cal (the state’s Medicaid program) to the undocumented population. Immigration-based exclusions were first removed for undocumented children in 2016, and further extended to young adults under 25 years old in January 2020. More recently, California expanded the state Medicaid program to those over 50 years old in May 2022 and the remaining adult population in 2024 [1]. The vast majority of undocumented immigrants meet the low-income requirements of Medicaid and collectively, the expansions brought down the uninsured rate among undocumented immigrants under 65 years of age in the state from 65% to 27% [2, 3].

This qualitative project examined the transition to full-scope Medi-Cal among newly eligible older undocumented patients in Southern California. We focus on the older adult expansion because it was the first large-scale expansion; over 359,000 older undocumented immigrants enrolled after the May 2022 policy [2]. Expanding health care access has been shown to improve health, utilization, and spending among older adults [4], underscoring the potential for the older adult expansion to positively impact the health and well-being of the undocumented population. While older adults in general are more likely to utilize all type of health care services [5], undocumented patients encounter multiple barriers to accessing healthcare, such as lack of transportation, limited English proficiency, and high costs, which often adversely influence help-seeking behaviors [6]. Other work has additionally suggested that undocumented immigrants avoid healthcare systems when they feel threatened by immigration enforcement [7]. It is unknown whether these barriers affected older undocumented immigrants’ transition to full-scope Medi-Cal and subsequent healthcare utilization.

We conducted a multi-modal qualitative study with focus groups and interviews with newly eligible older undocumented patients to understand their perspective in this significant policy shift. We aimed to answer the following questions: (1) How was this population getting their health needs met before Medi-Cal expansion? (2) What was the enrollment process like? (3) What have been their health care experiences after the policy change? We identified their medical needs and care pre-expansion, examined barriers and facilitators to the transition to Medi-Cal itself (i.e., learning about the policy change, and the enrollment process), continuity of care, changes in access and utilization, and changes in health status.

Our participants were patients from community clinics in two neighboring Southern California counties: Los Angeles and Orange counties. While Orange County has a smaller undocumented population (236,000) than Los Angeles County (951,000), the proportions of the foreign-born population that is undocumented are comparable (Orange: 25%, Los Angeles 28%) [8]. Prior to expansion, the counties varied in the available local healthcare resources for undocumented immigrants. Los Angeles County offered an indigent care program where undocumented immigrants were placed in a Federally Qualified Health Center (FQHC) primary care clinic and received emergency, specialty, and inpatient care at county hospital facilities at little or no cost [9]. In contrast, Orange County did not offer a local health coverage program and undocumented residents relied on fee-based episodic care at community clinics or emergency coverage through the federal Emergency (or “restricted”) Medicaid program. Emergency Medicaid was the only form of federal coverage for undocumented immigrants prior to Medi-Cal expansion and covers a limited set of medical emergencies (i.e., high risk pregnancy, acute psychiatric hospitalizations) [10]. Such differences in pre-expansion health care access are important considerations when examining the older undocumented population’s experiences with full-scope Medi-Cal.

To our knowledge, this is the first qualitative investigation into California’s Medi-Cal expansion. Our results offer important insight into how the program is being received in these communities and where barriers may still remain in ensuring optimal enrollment into Medi-Cal.

## Methods

### Sampling and recruitment

We used convenience sampling to recruit Latino patients who were newly eligible for full-scope Medi-Cal after the May 2022 expansion from two federally qualified health centers, one in Los Angeles, and the other in Orange County. While the clinics were primarily chosen based existing working relationships with the research team and our Steering Committee, the two clinics were in counties that varied in the health care access for undocumented immigrants prior to expansion. The Steering Committee included county healthcare officials, clinic directors, and Medi-Cal enrollers. We focused on Latino patients, as they are the largest undocumented population in the state and our research team had Spanish language capability. For both clinics, we were provided rosters of patients who qualified for the older adult expansion as determined by the clinics’ patient outreach programs. These patients were not eligible for Medi-Cal prior to expansion because of their immigration status and were 50 years of age or older on May 2022. We contacted individuals from the patient rosters by phone and text message using a Google Voice phone number specifically created for this project. When we reached potential participants over the phone, we introduced the study and screened individuals for eligibility if they expressed interest in participating. Our eligibility criteria were: (1) not enrolled in full-scope Medi-Cal prior to May 2022; (2) 50 years or older on May 2022; (3) Spanish or English speaking; and (4) undocumented status. Once eligibility was determined and participants expressed interest in the study, all offered verbal informed consent over the phone. For the focus groups, participants were then invited to the clinic at a certain date and time for the group discussion. For the individual interviews, the recruiter and participant decided on a convenient date and time for the phone interview. Participants were offered $40 gift cards to a local grocery for their participation.

The final sample was 12 individuals; two focus groups with six participants total, three interviews with Los Angeles patients, and three interviews with Orange County patients. We arrived at our final sample size through data and pragmatic considerations. First, we limited our data collection to the 2023 calendar year to ensure participants’ recall of the enrollment period and to discuss their health utilization patterns after being newly enrolled in Medi-Cal. We also wanted to avoid data collection after the final Medi-Cal expansion to all adults in January 2024. Other work has found that small sizes can be adequate in reaching saturation [11] and we believe we found saturation in several areas, which are presented in the results. The sample size is within the range of other qualitative studies with undocumented patients in health care setting [12–17]. We followed the Standards for Reporting Qualitative Research (SRQR) (Supplemental File 3) [18].

### Data collection

The focus groups were conducted in April 2023, the Los Angeles interviews were conducted between October and December 2023, and the Orange County interviews were conducted in September 2023 to December 2023. The focus groups and interviews were conducted in Spanish by a fluent facilitator/interviewer who had prior experience with Spanish-language qualitative data collection. The focus groups lasted approximately 1.5 h and each interview lasted approximately 45 min. Interviews and focus groups were audio recorded.

Both the focus groups and interviews used the same semi-structured guide. The interview questions were based on topics of interest identified by our Steering Committee as well as known factors that influence care-seeking among undocumented immigrants published in previous research [6, 19]. The guide covered participants’ medical needs and how they typically received care before enrolling in full-scope Medi-Cal and barriers to getting the care they needed; sources of information about Medi-Cal expansion leading up to the policy change; how they enrolled in Medi-Cal and whether they had any assistance; how they were assigned to their doctors under full-scope Medi-Cal and whether this was to their liking; what the process of getting medical care is like now with full-scope Medi-Cal; any changes in diagnosis or treatment since being on full-scope Medi-Cal; and any improvements or impediments to their health since being on full-scope Medi-Cal (see Supplemental File 1).

### Privacy and confidential information

Our study procedures were approved by UC Irvine's Institutional Review Board (IRB #1709). Because of the sensitive nature of immigration status, the patient rosters used for recruiting and participant lists were kept separately at all times. During data collection, all participants chose pseudonyms that were used throughout the focus groups and interviews so that the interviewers and other focus groups participants were unaware of actual names. The Spanish-language transcribers removed all identifying information before interview transcripts were translated to English.

### Data management and analysis

Audio recordings were transcribed into Spanish and translated to English by bilingual researchers. The eight transcripts were coded manually using thematic analysis. We chose thematic analysis because it can be used across a range of theoretical frameworks while also providing a systematic approach to data analysis through a coding scheme that identifies themes and patterns. It is also amendable to having multiple coders [20–22]. We took a combined deductive and inductive approach toward the data analyses of our study population. Our analysis progressed in four stages: (1) All co-authors reviewed the transcripts and wrote memos to familiarize themselves with the data. (2) We then indexed the transcripts into general themes that aligned with the study’s overarching research questions: existing medical needs and utilization prior to expansion, barriers and facilitators to enrollment, and changes in healthcare utilization and health status. This step represented the deductive part of our analysis. (3) We then inductively developed additional, analysis codes that characterized major issues identified by the participants under each theme. (4) To ensure internal reliability and validity, all co-authors met regularly to review and calibrate the inductive codes [23]. See Supplemental File 2 for the project codebook.

## Results

### Socio-demographic characteristics of participants

All 12 participants were born in Latin America, and the mean length of time in the US was 26 years (SD: 5.4; range: 15–31 years) (Table 1). The mean age of the participants was 58 years (SD: 5.9; range: 50–66 years). There were slightly more men (*n* = 7, 58%) than women (*n* = 5, 42%) in the sample. Spanish was primarily spoken in participants’ homes, and nearly all participants self-reported they spoke English “not well” or “not at all” (*n* = 10, 84%). Over half of the participants worked in production/warehouse, childcare, or housekeeping/maintenance on homes (*n* = 7, 58%), and the other half were not employed during the interviews (*n* = 5, 42%). The place of residence is the same as the county where the interview took place; nine participants resided in Los Angeles County and three in Orange County.

**Table 1:** Sample characteristics of focus group participants, *N* = 12

	*n*	%
**Biological Sex**		
Female	5	42%
Male	7	58%
**Age in years**		
Mean (SD)	58 (5.9)	
Min, Max	50, 66	
**Years lived in the USA**		
Mean (SD)	26 (5.4)	
Min, Max	15, 31	
**Language primarily spoken at home**		
Spanish	12	100%
**English Proficiency**		
Very good	1	8%
Good	1	8%
Bad	5	42%
Not at all	5	42%
**Marital Status**		
Single	2	17%
Married	9	75%
Widow/Widower	1	8%
Divorced/Separated	0	0%
**Education**		
Did not attend	1	8%
Primary School	6	50%
Secondary School	3	25%
University	2	17%
**Occupation**		
Unemployed	5	42%
Childcare	1	8%
Food Service	2	17%
Production/Warehouse	3	25%
Cleaning/Maintenance on Homes	1	8%
**Interested in keeping the same provider post-enrollment**		
No	0	0
Yes	12	100%
**In the past 12 months**,** how many times have you gone to a clinic for an illness?**		
0	2	17%
1	2	17%
2	1	8%
3	2	17%
4	1	8%
5 or more visits	4	33%
**How comfortable are you making medical appointments?**		
Very Comfortable	4	33%
Comfortable	7	58%
Neutral	1	8%
Uncomfortable	0	0%
Very Uncomfortable	0	0%
**Ever told you have diabetes? Not during pregnancy**		
No	4	33%
Yes	8	67%
**If has diabetes: Are you taking medications for it?**		
No	1	13%
Yes	7	88%
**Ever told you have hypertension?**		
No	6	50%
Yes	6	50%
**If has hypertension: Are you taking medications for it?**		
No	1	17%
Yes	5	83%
**Ever told you have heart disease**,** asthma**,** or cancer?**		
No	12	100%
Yes	0	0%
**In the past 12 months**,** have you ever felt you needed to see a professional for problems with your mental health**,** emotions**,** or substance use?**		
No	12	100%
Yes	0	0%

Eight participants (67%) reported being diagnosed with pre-diabetes or type 2 diabetes (T2D), and six (50%) reported having hypertension. Nearly all participants were taking medication for their chronic conditions (taking T2D medication *n* = 7, 88%; taking hypertension medication *n* = 5, 83%). None of the participants had ever been diagnosed with asthma, heart disease, or cancer, and none perceived a need for mental health or substance use treatment in the past year. Most participants were comfortable scheduling medical appointments (*n* = 11, 92%). There was no clear pattern in the frequency of medical visits for illnesses in the past year. All participants wanted to stay with the same provider after enrolling in full-scope Medi-Cal.

### Getting medical needs met before Medi-Cal

Prior to Medi-Cal expansion, most of the participants had some coverage through restricted, or emergency, Medi-Cal, which offers coverage for certain conditions and emergency care. However, many participants noted the lack of full coverage under restricted Medi-Cal and commented on its narrow scope of emergency care. Participants from Los Angeles County who were enrolled in MyHealth LA (MHLA), the local health coverage program in Los Angeles County, reported much better access to comprehensive care. These participants had established relationships with a primary care doctor - even those who had advanced chronic conditions that required intensive medical care, such as T2D. People enrolled in MHLA were generally satisfied with their care pre-expansion and were able to be seen by their doctors regularly, received prescriptions, and saw specialists for their conditions. Cesar, a 50-year-old focus group participant from Los Angeles, had regularly seen both his primary doctor and specialists for his prostate condition for the previous three years at no cost.

*I and I think other people have been very satisfied with the MyHealthLA plan in the sense of the care it provides to people in need like me and many others. […] The service that has been provided to people is excellent and it has been good in the sense that we have had access to see a doctor. We have had access to go to the hospital when necessary and we have also had access to medicines…. I have been under the care of MyHealthLA and have been seeing specialists at the county hospital for three years. So in three years I haven’t received a charge.*

However, even those who had regular health care through MHLA reported having to pay for some services. Dental services came up regularly as a medical expense before full-scope Medicaid. Maricela, a 53-year-old focus group participant, described being generally happy with her MHLA care but when she tried to see a specialist dentist for more complicated dental work, she was told she would have to pay for services.

*No*,* well the doctor who was seeing me here [the primary care clinic]*,* he did my [dental] cleaning and tooth fillings. But when he couldn’t do what he had to do with my teeth*,* he sent me to [a university clinic]. There they charged me about $500 to do a deep cleaning. I went to have my nerve killed and my nerve wasn’t good afterwards. So they did it again*,* but then the doctor told me “Here you have to pay*,* the insurance you have does not cover it”.*

Not all participants had access to regular comprehensive care prior to Medi-Cal expansion. We especially saw this from our Orange County participants whose healthcare utilization history prior to Medi-Cal expansion differed widely amongst one another. While some participants reported visiting the local FQHC and paying on sliding cash scale, this type of care was more sporadic and participants were concered about costs. Carlos, a 57-year-old interview participant from Orange County, described how these costs were initially manageable but could quickly escalate as medical needs became more complex. Yet paying for services did not guarantee a positive outcome or sound medical advice.

*At the clinic called [name]*,* they started treating me there for high blood pressure. The consultation was $25. But once I was there*,* since my blood pressure was very high*,* they gave me an electrocardiogram*,* which was $180 and then a blood test which was $150. So total $350. The next time I went they said again “Your blood pressure is still very high and you have to go see a cardiologist.” That was $1*,*500 dollars. And when I got to the cardiologist they told me*,* “The truth is your heart rate is fine*,* maybe your electrocardiogram was wrong. To me*,* your heart rate is fine”.*

Francisco, a 62-year-old male interview participant from Orange County, reported visiting the community clinics for certain health conditions, despite having employer-based insurance. These findings underscore the importance of community-based clinics for undocumented patients in areas where local healthcare coverage is not available. While these fee-based clinics were the main source of their medical care, concern over cost encouraged participants to seek care primarily for acute conditions rather than regular maintenance of chronic conditions.

### Enrolling in full-scope Medi-Cal

Participants had heard about the state Medi-Cal expansion policy through their providers, family, and the television. However, these sources were secondary compared to the official letter from the state Medi-Cal office that officially confirmed their eligibility status and provided information on how to enroll. Maria, a 50-year-old focus group participant, said the letter was significant above other sources of information.

*On TV they announced that undocumented people were already going to have regular Medi-Cal. And well I say*,* if I don’t see it*,* I don’t believe it. And then I received that paper saying your Medi-Cal is regular*,* it’s not like before.*

While other work has suggested that complicated paperwork can deter healthcare utilization among undocumented Latino patients [24], the respondents in our study consistently mentioned the official Medi-Cal documentation from the state as an important factor in their enrollment. One key component of the letter, however, was contact information for enrollment support. While the letter held symbolic meaning for eligibility, it also provided useful resources that the pariticipants relied on to clarify the enrollment process. Hernan, a 58-year-old focus group participant, spoke of actively contacting the resources provided on the letter to select a primary care physician.

*They sent us [the letter] and a phone number to call for a provider to attend to us and explain what types of insurance or what insurance company is going to provide us with the service. […] Well*,* at least in my case*,* they called me from the clinic here*,* then they sent me the letter and I had to call the county to have a provider tell me about the Medi-Cal benefits*,* what types of insurance they have and what clinics they work with.*

Participants overwhelmingly received help in enrolling for Medi-Cal, primarily from the county Medi-Cal office, social workers at community clinics, or family members. The lone participant who reported trying to enroll online ran into technical problems and ultimately remained unenrolled at the time of the interview. While the participants could not always recollect who enrolled them and which organization they were affiliated with, their reliance on others emphasizes the importance of support staff and resources to enroll newly eligible patients. Carlos a 57-year-old interview participant from Orange County recounted how he continued to rely on the social workers who helped him enroll to navigate additional questions about his health coverage.

*I even spoke to [the social worker] yesterday because I told her that they’ve been sending me bills for the time I was hospitalized because I haven’t received the card*,* and she tells me*,* “Look*,* I have the card here*,* I’m going to give it to you. If they talk to you again*,* give them the card number. What I’m seeing here in the system is that yes*,* they did qualify you and you already have Medi-Cal*,* but starting in October. So*,* right now I’m going to send an email*,* asking why starting in October*,* since the paperwork that was sent in included August and September*,* which is when you were hospitalized. Let’s see what they answer and I’ll contact you to let you know. For whatever reason if you get papers in the mail*,* and I’ll assist you”.*

It is also notable that many of the participants like Carlos who were actively seeking care received information about Medi-Cal expansion directly from their health care providers or clinic staff. First, this highlights the importance of healthcare providers in enrolling newly eligibxle patients. Second, this also implies that the first waves of newly eligible individuals who enroll may be those who have ongoing conditions and have regular contact with care providers. Undocumented patients who are removed from the health care system may require other forms of outreach.

Interestingly, none of the participants discussed immigration concerns as a deterrent from enrolling. While other research suggests that undocumented immigrants are fearful when interacting with bureaucratic entities like the health care system [25], the participants in our study denied that immigration concerns weighed into their decision to enroll. Hernan, a 58-year-old Los Angeles focus group participant, was reassured by clinic staff that his application to Medi-Cal would not affect his status.

*So*,* they explained to us that it would not affect us at all and that there was already a law that we could have the complete Medi-Cal that covered us who are 50 years and older. And well*,* fear to not do it is mainly because one’s status. But as they explained to us that it does not harm us at all then so we already feel more confident in doing the process.*

While the majority of our participants were able to enroll successfully, there was a minority who were not enrolled at the time of the interview. One interview participant, Javier, a 50-year-old from Los Angeles, had tried to enroll but was repeatedly denied for reasons that were unclear to him. Despite receiving assistance from workers at the health clinic and the county Medi-Cal office as well as submitting additional information, his application was denied. The consequences were dire, as he had Type 2 Diabetes and was not able to access needed medication.

*They [the clinic] really tried to help me. Myriam would call me almost daily*,* asking what happened. […] She tried to help me so that they would give me the Medi-Cal*,* but ultimately*,* she told me that I just needed to wait and they couldn’t do anything else. It’s been more than a year since I have had medical assistance. I don’t have medicine. The truth is…I’m not lying to you or exaggerating that there are people who have diabetes and take the same pill and they sell the pills to me.*

Even though it was rare to have applications denied, these rejections had major repercussions. The health consequences were compounded by the individuals’ confusion and lack of clarity on the reasons for their rejections, even as they underwent substantial administrative burden to prove their eligibility.

### Health care experiences after Medi-Cal expansion

Participants who had regular care through MHLA prior to expansion saw few differences after Medi-Cal expansion, even wanting to stay with the same primary care providers to minimize any care disruptions. Maricela, a 58-year-old focus group participant, even went so far as to call the Medi-Cal county office to switch back to her original doctor after she was assigned to another medical network. Maria, a 50-year-old Los Angeles focus group participant, explained that having a new physician would be like starting over and that she would prefer staying with the same doctor, even if it was further away.

*[…] I will have to fill out new paperwork at another clinic and another doctor will not know what I have. Here they already have my file*,* they know what I have and how I am controlling it. Well*,* it’s hard for me to see myself anywhere else. Even if it is close to my house*,* I won’t change clinics.*

The participants specifically cited having accessible schedules and Spanish-speaking staff as important features they valued in their providers.

While MHLA participants were able to maintain their primary care providers according to their preferences, they did experience some disruptions in their specialty care. Prior to Medi-Cal expansion, their specialist doctors were affiliated with the county health system through the MHLA program. Once they were on full-scope Medi-Cal, however, they often found that these county doctors were no longer taking their new health insurance and they had to be referred to new specialists. Esmeralda, a 63-year-old focus group participant, found out that she could not see an ophthalmologist that she had already made an appointment for because they didn’t take her new insurance. Her primary care clinic was able to refer to her another ophthalmologist practice where her new insurance plan was accepted.

*I already had my appointment for my eyes. But they said*,* “No*,* I can’t take care of you anymore. You have to switch to this hospital’s plan.” I tell them no because it is far away. I tell him I’m not going there anymore*,* and it’d be better for me to wait. I came here [the primary care clinic] with my same doctor [her primary care physician]*,* and I told him that they [the ophthalmologist] longer wanted to treat me. He says*,* “Don’t worry. We’re going to find you another place where you can go.” And thank God that they already sent me to that hospital [a new specialty clinic] where they are treating my eyes.*

For participants without regular access to care prior to the change, the impact of Medi-Cal expansion was primarily felt in their reduced medical costs. Jorge, a 58-year-old Orange County interview participant, described two healthcare encounters, one prior to Medi-Cal expansion and one after, that illustrated the financial impact of having Medi-Cal coverage. Prior to Medi-Cal expansion, Jorge was uninsured and visited the ED for dizziness. While he believed he had health coverage at the time, the hospital informed him that his insurance was invalid and the visit ended up costing him $1,300, which he paid off in installments. After enrolling in full-scope Medi-Cal, he visited the ED again, this time for arm pain, and received a bill for $1900. He called Medi-Cal staff who explained that his insurance would be covering the cost.

*That person told me that my 2022 card was activated. The card now covered all my costs completely. The medication*,* anything that I have was going to be covered. […] I felt a relief because God was there with me. A bill of $1*,*900*,* how could I pay it without money?.*

Some of the participants discussed changes in utilization as well, as some were able to have medical procedures that were previously not covered by Emergency Medicaid. Rosa, a 63-year-old Los Angeles interview participant, was able to have cataract surgery after she enrolled in full-scope Medi-Cal. Emergency Medicaid alone could not address all of her medical needs but full-scope offered a wider array of treatment options for her surgery. As a result of this expanded access, Rosa was able to have her needed surgery.

*Ok*,* before it was just the emergency MediCal and it’s not like the other one [full-scope MediCal]*,* like it wasn’t very good. Because it’s not like the one we have right now*,* that cover the cataracts*,* the surgeries and all that. Yes*,* it is better now than before.*

All participants, regardless of the tangible changes that full-scope Medi-Cal offered, described achieving greater peace of mind about keeping medical costs low which was missing under Emergency Medicaid and even MHLA. Hernan acknowledged that he had no medical costs prior to enrolling in full-scope Medi-Cal because of MHLA. However, having full-scope Med-Cal offered the perception of more stable coverage.

*No*,* no*,* not before [he didn’t pay for medical costs before]. But there was the uncertainty of when are they going to take it from me*,* when will they no longer be able to cover my medical needs. Now no. Now I have medical service and I have service*,* medicine and all that*,* so no*,* that is not a concern right now. There is a change in benefits.*

This newfound security in healthcare access could lead to more active healthcare utilization that patients may have foregone in the past, even with MHLA coverage. Maria, the focus group participant, described being more willing to go to the hospital with full-scope Medi-Cal because of the assurance that the program would cover the costs.

*Well*,* if they ask me*,* “Are you going to the hospital?” I’m going to go with more confidence*,* because if before I was dying*,* I said I’m not going because I’m going to get a bill of $3*,*000*,* $2*,*000. And I am afraid. Where am I going to get that money? How am I going to pay it?.*

However, participants still expressed uncertainty over what exactly is covered on the full-scope and wished to find out the extent of full-scope Medi-Cal coverage. Caesar, the focus group participant wanted to have more information on what is covered and his expected payments for medical care.

*For me the most practical thing would be to have all the corresponding information. Or a little deeper understanding into what exactly the program is going to cover in the sense of what should I pay*,* what should I not pay? What specialist is covered by this program? Or what specialist is not covered by this program? What medication is covered by this program or what medication is not covered by this program? Or for me it would simply be to deepen my knowledge of the program a little more.*

While some participants were actively looking for information, others did not express concern over understanding their new benefits. Alma, a 66-year-old Los Angeles acknowledged that her lack of information about full-scope Medi-Cal would not affect how she would interact with her health care providers.

*I’m very stubborn and nothing sticks with me. And they told me what kind of MediCal insurance I had*,* but I don’t even remember which one I have. I go to the doctor*,* and they treat me and I don’t even know what insurance company I have.*

## Discussion

Our study sought to understand multiple aspects of the transition to Medi-Cal for undocumented older adults in Southern California. We studied the health needs of this population, the barriers and facilitators to enrollment in full-scope Medi-Cal, and potential changes in health care utilization and health status due to their transition to full-scope Medi-Cal. To our knowledge, this is one of the first studies to explore the older adults’ Medi-Cal expansion among undocumented patients in California.

All participants were pleased with full-scope Medi-Cal, even if they did not immediately see changes in their healthcare utilization and health status. Participants expressed fewer financial worries about seeking healthcare services and an increased sense of emotional security that they would not lose access to healthcare. This was true even for participants who had regular access to care pre-expansion, since full-scope Medi-Cal was perceived as more permament and stable than the local program that pre-dated the expansion. For participants who had irregular care, the expansion signified increased access to healthcare services and a significant reduction in healthcare expenses since they mainly paid for medical services out of pocket. Many of the participants reported plans of seeking specialty care in the future.

However, participants reported not being fully aware of all the services they were newly eligible for. Eligible patients may need additional assistance in understanding what new services they can access to facilitate changes in their utilization patterns. The sense of emotional security could encourage more healthcare utilization to manage chronic health conditions and prevent the development of other diseases.

Most of the participants successfully enrolled in full-scope Medi-Cal, which is a promising indicator of the reach of the policy in expanding access to care. However, this comes with the important caveat that nearly all of the participants required outside assistance in enrolling. One participant who tried to enroll by himself was unsuccessful in navigating the website and conceded that he would look for enrollment support. This finding points to the critical importance of clinic staff at FQHCs and Medi-Cal enrollment staff to ensure that patients enroll in the program and successfully renew annually. While the application for full-scope Medi-Cal can be completed online, undocumented immigrants and older Latinos often have difficulty accessing computers and the internet and have limited digital literacy [26]. Our participants were also FQHC patients; clinics often have enrollment support processes set up for uninsured patients, including multilingual staff and social resources. This may also explain participants’ low concern about their legal status in enrolling in Medi-Cal. Providers who regularly treat undocumented patients may make efforts to create welcoming environments, provide resources and health navigators for social needs, and provide trauma-informed care [27]. Our research aligns with others that has found linguistically and culturally competent staff and health navigators to be important facilitators of care [25].

Finally, our results suggest that the experience of transitioning to full-scope Medi-Cal was shaped by the kind of health care access individuals had before the expansion. In Los Angeles County, undocumented patients had access to a safety net through the county health coverage program, whereas undocumented patients in Orange County paid steep prices for medical care and used it sporadically. Patients who had more regular health care utilization prior to expansion experienced little disruption in their primary care and all stated a strong preference to remain with the same providers. However, some mentioned difficulties getting affordable dental care prior to expansion and saw changes in their specialty care post-expansion. Orange County participants experienced major differences in the cost of care before and after Medi-Cal expansion. The local health care environment could be an important factor moderating the impact of the expansion and future research could consider regional differences.

### Limitations and implications

We acknowledge limitations in our study. First, we only recruited patients from two FQHCs, one in Los Angeles County and one in Orange County.The small number of clinics and the small number of counties may limit the generalizability of our findings to undocumented immigrants living in other parts of the state. Our participants only came from two out of the 58 California counties and are in Southern California, primarly urban, and have a large immigrant population. As such, we cannot claim that our sample is representative of the newly eligible Medi-Cal population statewide. We therefore view our results as starting off points for future research on this major policy change.

There was also a difference in sample size between the Los Angeles and Orange County participants. The small number of Orange County patients precludes us from making any concrete conclusions on regional differences in the impact of the expansion. Moreover, since these patients were already in contact with the healthcare system, their experiences may not be representative of undocumented adults who avoid seeking healthcare services, including hard-to-reach populations such as seasonal labors or newly arrived immigrants. However, this is still an important segment of the undocumented population to study, as they are the people most likely to maintain their enrollment in Medi-Cal. Nonethelss, we acknowledge that we are missing older undocumented immigrants who are not in regular contact with the formal health system and future research could identify important barriers and facilitators to enrollment among these patients. We also lacked enough female participants to do gender comparisons. Nonetheless, the Los Angeles County focus groups had roughly equal numbers of women and men, with no apparent gender differences in their experiences. Finally, we also acknowledge that the focus groups were conducted only with Los Angeles County residents. This could have influenced the results if the group setting deterred the discussion of sensitive topics, such as the role of their immigration status on enrollment. However, the consistency of the findings on this topic across both the focus groups and the interviews suggests that the medium of data collection did not play a role in this finding. For both modalities, participants denied that concerns over their immigration status deterred enrollment.

## Conclusions

Our study explored patient experiences in the transition to Medi-Cal for older undocumented immigrants through focus groups and interviews. To our knowledge, this is the first study of its kind and our findings offer several important insights into California’s Medi-Cal expansion that deserve future study. We found that enrollment was high and patients viewed their experiences in full-scope Medi-Cal positively. While some did not immediately notice changes in their health care, others experienced substantial drops in medical costs. Our findings emphasize the need for clinic staff and Medi-Cal enrollment staff to support successful enrollment and renewal for this population. Qualitative research should continue to explore the role of social services as facilitators for enrollment and identify additional barriers to care in this vulnerable population. Future research should investigate whether these findings were relevant for older undocumented adults living in different areas of the state or in counties with a smaller immigrant population. Finally, our findings suggest that future quantitative analysis of Medi-Cal expansion should consider differences in the policy impact by county.

### Researcher positionality statement

All researchers have advanced degrees, some first generation college students, and are interested in the health and health care utilization of immigrant, monolingual populations. Some of the research team are of Latino descent and have personal experiences navigating the immigration and health care systems in Southern California.

## Supplementary Information

Below is the link to the electronic supplementary material.

Supplementary Material 1

Supplementary Material 2

## Data Availability

The datasets generated and/or analyzed during the current study are available from the corresponding author on reasonable request.

## Abbreviations

T2D: Type 2 diabetes
FQHC: Federally Qualified Health Center
LA: LA County
MHLA: MyHealth LA
