# Supplementary material for: A qualitative study on the transition to full-scope Medi-Cal among older Latino patients in California
Source: BMC Health Serv Res. 2025 Dec 31;26:166. doi: 10.1186/s12913-025-13961-6 (PMC12865940; doi:10.1186/s12913-025-13961-6)
Supplement: Supplementary file 1 — Supplementary Material 1 [file 12913_2025_13961_MOESM1_ESM.pdf]

Focus Group Questions – Enrolled Patients

Facilitator: In May 2022, California made everyone 50 years and older who meet income requirements eligible for Medi-Cal. Immigration status does not matter. This means that you have health insurance through Medi-Cal. We've invited you all here because you are newly eligible for full-scope Medi-Cal and we wanted to understand how that experience is.

Before we start, I want to explain what I mean when I say full-scope Medi-Cal. Some of you may have had "restricted" or "emergency" Medi-Cal, which is a type of health coverage for low-income individuals who did not qualify for Medi-Cal before May 2022 because of the citizenship requirements. Emergency Medi-Cal only offers limited coverage for health conditions. Full-scope Medi-cal, which you are now fully eligible for, covers more than just care when you have an emergency. It provides medical, dental, mental health, and vision (eye) care. It also covers alcohol and drug use treatment, drugs your doctor orders, and more. All services are for free or for low-cost.

1. Welcome and Introductions. Can you introduce yourself (name and age) and how long you have been coming to [UCI/Eisner Health]? [TOPIC: Introductions]

2. We'd first like to understand your current medical needs. Do you have any ongoing health issues? Like high blood pressure or diabetes?

3. Thinking about BEFORE May of this year, can you tell me how you usually got medical care? [TOPIC: Usual medical care access and utilization before Medi-Cal expansion and any chronic health conditions]
    a. How did you find your doctor?
    b. Did you have one or more doctors that you saw regularly (such as primary care doctor, nurse practitioner or Physician Assistant)? Who were they and what kind of doctors were they?
        i. Probe: specialty care doctors such cardiologist, physical therapists or non-primary care doctors?
    c. Can you tell us about any times you had a problem getting the medical care that you needed?

4. How did you learn that you were eligible for Medi-Cal? [TOPIC: What kind of information did patients receive about the policy change and how helpful it was for them]
    a. Did you receive any information (mail, any in person information, calls, texts, TV, radio news) from anyone about this change before it happened?
        i. What did you receive?
        ii. How helpful was this information for you?
    b. Did anyone hear about it from family or friends? What did you hear from them?
    c. Did anyone else have a similar experience? Did anyone else have a different experience?

5. Do you know what new services you can get now with full-scope Medi-Cal ? [TOPIC: What patients know about full-scope and how it differs from their previous health coverage]
    a. How did you receive that information?

6. How did you enroll in Medi-Cal? Did anyone help you with enrollment? [TOPIC: Enrollment: experiences with enrollment: common ways that patients enrolled and any barriers in the process]
    a. What type of assistance were you offered when enrolling in Medi-Cal? Ex: did someone help you fill out the application, were you offered translation services?
    b. Did you encounter any problems enrolling?
    c. Did you have any fears or worries about enrolling?
        i. Did anyone have any concerns related to their immigration status? Why or why not?
        ii. Affect on other government services? Why or why not?
        iii. Affect on the health care you were already receiving? Why or why not?
    d. If there were concerns, what made you decide to enroll?

7. When you're on Medi-Cal, patients have to choose a primary care doctor or get assigned to one. [TOPIC: Continuity of care and choosing networks]
    a. How did you choose your primary care physician?
        i. Did you choose or were you assigned to one?
        ii. Were you able to keep the same doctor after enrolling in Medi-Cal?
        iii. Is keeping the same doctor that you had before Medi-Cal important to you? Why or why not?

8. Have you tried to see a doctor since being on Medi-Cal? [TOPIC: Utilization post-enrollment]
    a. What was that process like to get an appointment?
    b. For those of you with a new primary care doctor, how has that change been for you?
    c. Have any of your specialty care doctors changed since being on Medi-Cal? What has that change been like? [do they like the doctors more, do they prefer the location, etc?]
    d. Have you had any new prescriptions?
    e. Any new diagnoses?
    f. Is there anything you'd like to do for your health now that you have full-scope Medi-Cal?

9. What is better in terms of your health care now that you have Medi-Cal? What is worse?

Focus Group Questions – Eligible, but not Enrolled Patients

Facilitator: In May 2022, California made everyone 50 years and older who meet income requirements eligible for Medi-Cal. Immigration status does not matter. This means that you have health insurance. We've invited you all here because you are newly eligible for full-scope Medi-Cal and we wanted to understand how that experience is.

Before we start, I want to explain what I mean when I say full-scope Medi-Cal. Some of you may have had "restricted" or "emergency" Medi-Cal, which is a type of health coverage for low-income individuals who did not qualify for Medi-Cal before May 2022 because of the citizenship requirements. Emergency Medi-Cal only offers limited coverage for health conditions. Full-scope Medi-cal, which you are now fully eligible for, covers more than just care when you have an emergency. It provides medical, dental, mental health, and vision (eye) care. It also covers alcohol and drug use treatment, drugs your doctor orders, and more. All services are for free or for low-cost.

1. Welcome and Introductions. Can you introduce yourself (name and age) and and how long you have been coming to [UCI/Eisner Health]? [TOPIC: Introductions]

2. We'd first like to understand your current medical needs. Do you have any ongoing health issues? Like high blood pressure or diabetes?

3. Thinking about BEFORE May of this year, can you tell me how you usually got medical care? [TOPIC: Usual medical care access and utilization before Medi-Cal expansion and any chronic health conditions]
    a. How did you find your doctor?
    b. Did you have one or more doctors that you saw regularly (such as primary care doctor, nurse practitioner or Physician Assistant)? Who were they and what kind of doctors were they?
        i. Probe: specialty care doctors such cardiologist, physical therapists or non-primary care doctors?
    c. Can you tell us about any times you had a problem getting the medical care that you needed?

4. Did you know that you are eligible for Medi-Cal before coming to this group today? Of those who knew, how did you learn that you were eligible for Medi-Cal? [TOPIC: What kind of information did patients receive about the policy change and how helpful it was for them]
    a. Did you receive any information (mail, any in person information, calls, texts, TV, radio news) from anyone about this change before it happened?
        i. What did you receive?
        ii. How helpful was this information for you?
    b. Did you anyone hear about it from family or friends? What did you hear from them?
    c. Did anyone else have a similar experience? Did anyone else have a different experience?

5. Do you know what new services you can get now with full-scope Medi-Cal ? [TOPIC: What patients know about full-scope and how it differs from their previous health coverage]
    a. How did you receive that information?

6. Have any of you tried to enroll in Medi-Cal? [TOPIC: Enrollment: experiences with enrollment: common ways that patients enrolled and any barriers in the process]
    a. Can you tell us about that experience?
    b. Why didn't you end up enrolling?

7. For those of you who have not tried to enroll yet what has kept you from enrolling? [TOPIC: Barriers to enrollments for those who are not enrolled]
    a. Did anyone have any concerns related to their immigration status? Why or why not?
    b. Affect on other government services? Why or why not?
    c. Affect on the health care you were already receiving? Why or why not?
    d. If there were concerns, what helped you get over them?

8. How likely do you think you will enroll in Medi-Cal in the future? Why? [TOPICS: Addressing potential barriers]
    a. What would help you enroll in Medi-Cal?

11. When you're on Medi-Cal, patients have to choose a primary care doctor or get assigned to one. [TOPIC: Continuity of care and choosing networks]
    a. Is keeping the same doctor important to you? Why or why not?

12. What will you do to address your health concerns in the future if you are not on Medi-Cal? [TOPIC: Future care use among non-enrolled]
