# Supplementary material for: A qualitative study on the transition to full-scope Medi-Cal among older Latino patients in California
Source: BMC Health Serv Res. 2025 Dec 31;26:166. doi: 10.1186/s12913-025-13961-6 (PMC12865940; doi:10.1186/s12913-025-13961-6)
Supplement: Supplementary file 2 — Supplementary Material 2 [file 12913_2025_13961_MOESM2_ESM.docx]

**Codebook**

| **Index Codes** | **Analysis Codes** |
| --- | --- |
| Existing medical needs and utilization prior to expansion | MHLA patients were seen by doctors and did not have to pay |
|  | Accessing care from a general practitioner or specialist with coverage pre-expansion |
|  | Lapses in coverage or paying out of pocket |
|  | Enrollment in Emergency Medi-Cal |
| Barriers and facilitators in enrollment | Patients with regular contact were told clinics that they were eligible for full scope |
|  | Received notification by mail, media (television, radio), and clinic |
|  | Relying on help in navigating healthcare systems |
|  | No perceived barriers to enrollment |
|  | No concerns about their documentation status when enrolling |
|  | Successful enrollment in full-scope Medi-Cal with help |
|  | Technical difficulties |
|  | Not able to qualify/Has not enrolled |
| Changes in health care utilization and health status | No desire to change doctors |
|  | Wanting accessibility of care |
|  | Finding a doctor that takes their plan |
|  | Feeling more secure |
|  | Needing clarification on what is covered |
|  | Still having problems with coverage |
|  | Problems not related to coverage, but complaints about accessing care (e.g., getting appointments, transportation) |
|  | Wanting to take care of different health problems |
